# 获得性凝血因子X缺乏症1例报告并文献复习

**DOI:** 10.3760/cma.j.cn121090-20241130-00512

**Published:** 2024-12

**Authors:** 婧 张, 葳 刘, 玉华 王, 仁池 杨, 磊 张, 峰 薛

**Affiliations:** 1 中国医学科学院血液病医院（中国医学科学院血液学研究所），血液与健康全国重点实验室，国家血液系统疾病临床医学研究中心，细胞生态海河实验室，天津 300020 State Key Laboratory of Experimental Hematology, National Clinical Research Center for Blood Diseases, Haihe Laboratory of Cell Ecosystem, Institute of Hematology & Blood Diseases Hospital, Chinese Academy of Medical Sciences & Peking Union Medical College, Tianjin 300020, China; 2 天津医学健康研究院，天津 301600 Tianjin Institutes of Health Science, Tianjin 301600, China; 3 中国医学科学院北京协和医学院群医学及公共卫生学院，北京 100730 School of Population Medicine and Public Health, Chinese Academy of Medical Sciences and Peking Union Medical College, Beijing 100730, China

**Keywords:** 凝血障碍, 出血, 获得性凝血因子缺乏, 原发性轻链型淀粉样变, Coagulation disorders, Bleeding, Acquired coagulation factor deficiency, Primary light-chain amyloidosis

## Abstract

**目的:**

介绍获得性凝血因子Ⅹ缺乏症的临床特征、实验室检查、诊断思路及治疗。

**方法:**

回顾1例获得性凝血因子Ⅹ缺乏起病的原发性轻链型淀粉样变患者的诊疗过程并进行文献复习。

**结果:**

患者为69岁男性，因腹腔出血入院，经检查确诊为原发性轻链型淀粉样变合并获得性凝血因子Ⅹ缺乏症，反复出血是因为沉积在外周血管及脏器中的淀粉样物质吸附了循环中的凝血因子Ⅹ，使其沉积在网状内皮系统（如脾脏），导致凝血因子Ⅹ水平下降。患者经积极止血及原发病治疗后出院。

**结论:**

该病例为临床上罕见获得性凝血因子缺乏症提供了诊疗思路及借鉴。

## Abstract (en)

**Objective:**

To introduce the clinical features, laboratory findings, diagnostic approaches, and treatment of acquired factor X deficiency.

**Methods:**

This study reviewed a case of primary light-chain amyloidosis with acquired factor X deficiency admitted to our hospital. The patient's clinical course and diagnostic process were described, and a literature review was conducted.

**Results:**

The patient, a 69-year-old male, was admitted to our hospital due to abdominal bleeding. He was diagnosed with primary light-chain amyloidosis complicated by acquired factor X deficiency. Recurrent bleeding was attributed to amyloid deposits in peripheral blood vessels and organs, which adsorbed circulating factor X, causing its sequestration in the reticuloendothelial system（e.g. the spleen）and leading to decreased factor X levels. After active hemostasis and treatment of the underlying disease, the patient was discharged.

**Conclusion:**

This case provides insights and a reference for the diagnosis and management of the rare clinical presentation of acquired coagulation factor deficiency.

凝血因子X（FX）作为内源性和外源性凝血途径共同的关键凝血因子，经激活后与Ca^2+^、磷脂及凝血因子Ⅴ（FⅤ）共同形成复合物，后者最终使凝血酶原激活为凝血酶。获得性FⅩ缺乏症是指其他疾病导致的FX缺乏症，常继发于原发性轻链型淀粉样变（pAL）[1]。患者实验室检查表现为活化部分凝血活酶时间（APTT）及凝血酶原时间（PT）延长、FⅩ活性（FⅩ∶C）减低等。临床症状多表现为皮肤、黏膜出血，也表现为肌肉血肿、血尿、消化道出血等。治疗包括急性出血的替代治疗和原发病的治疗。本文报道1例以腹腔出血表现起病的pAL伴获得性FⅩ缺乏症的病例并进行文献复习。

## 病例资料

患者男性，69岁，主因“反复皮肤瘀斑1年，剑突下不适1月余，伴乏力11 d”于2020年4月16日入我院。该患者入院前1年无明显诱因反复出现四肢皮肤瘀斑，未予特殊重视。入院前1月余，患者突发剑突下撕裂样疼痛，呈固定性持续性疼痛，遂就诊于当地医院，查腹部MRI提示腹腔出血，腹部穿刺抽出不凝血，当地医院予患者血浆、红细胞输注，抗感染等治疗，出血控制后出院。入院前11 d，患者因乏力头晕再次就诊于当地医院，测血压80/50 mmHg，当地医院紧急补液、血浆输注等治疗后转诊于我院，门诊血常规HGB 108 g/L，凝血功能：APTT 47 s（参考值23.7～36 s），PT 31 s（参考值10～14 s），考虑患者凝血功能异常。既往史、个人史、家族史均未见特殊。体格检查提示贫血貌，肝肋缘下1.5 cm。

入院后完善相关检查，血常规：RBC 3.62×10^12^/L，HGB 100 g/L，红细胞压积（HCT）30.1％，红细胞生成素（EPO）28.46 mIU/ml（参考值2.59～18.50 mIU/ml），网织红细胞（RET）3.76％（参考值0.50％～1.50％）。总胆红素（TBil）29.3 µmol/L（参考值0～260 µmol/L），直接胆红素（DBil）10.3 µmol/L（参考值0～4.0 µmol/L），间接胆红素（IBil）19.0 µmol/L（参考值0～22.0 µmol/L），γ-谷氨酰转移酶（GGT）60 U/L（参考值8～57 U/L），碱性磷酸酶（ALP）158.8 U/L（参考值30.0～120.0 U/L），乳酸脱氢酶（LDH）209.8 U/L（参考值0～247.0 U/L），尿素9.13 mmol/L（参考值2.80～7.60 mmol/L），肌酐（Cr）83.4 µmol/L（参考值64.0～104.0 µmol/L），尿酸（UA）424.9 µmol/L（参考值208.3～428.4 µmol/L），血糖5.47 mmol/L（参考值3.90～6.10 mmol/L）。免疫全项：IgA、IgG、IgM、C3、C4、C反应蛋白均未见异常，抗核抗体阳性1∶1000，狼疮抗凝物阴性，抗磷脂抗体阴性。凝血功能：PT 31.3 s，国际标准化比值（INR）2.90（参考值0.87～1.20），APTT 40.7 s，凝血酶时间（TT）16.2 s（参考值13.3～19.3 s），纤维蛋白原3.24 g/L（参考值2.00～4.00 g/L）。脑钠肽（BNP）362 ng/L（参考值0～100 ng/L），尿常规示潜血+、蛋白2+、尿胆原3+，24小时尿蛋白0.6105 g。甲状腺功能、肿瘤全项、病毒、血小板功能、LGL、便常规均未见异常。FV∶C 39.6％（参考值50.0％～120.0％），FⅩ∶C 6％（参考值50％～120％）。Bethesda法检测FX抑制物0 BU/ml。1∶1血浆纠正试验：PT及APTT即刻和37 °C孵育2 h均可完全纠正。为明确FⅩ缺乏原因，行凝血酶原复合物（PCC）回收率实验，输注PCC 3000 U（50 U/kg）30 min后复查FⅩ∶C未见升高（输注前APTT 47 s，PT 31 s，FⅩ∶C 7.6％；输注后APTT 46 s，PT 21.2 s，FⅩ∶C 11.8％）。血免疫固定电泳未见M蛋白，轻链κ 7390 mg/L（参考值6290～13500 mg/L），轻链λ定量4130 mg/L（参考值3130～7230 mg/L），κ/λ比值1.78（参考值1.53～3.29）；游离轻链κ（FLC κ）19.9 mg/L（参考值3.3～19.4 mg/L），游离轻链λ（FLC λ）147.0 mg/L（参考值5.7～26.3 mg/L），κ/λ比值0.14（参考值0.26～1.65），β2微球蛋白4.42 mg/L（参考值1.09～2.53 mg/L）；尿免疫固定电泳：在γ区可见一条单克隆轻链λ成分，FLC κ 80.7 mg/L（参考值0～18.5 mg/L），FLC λ 141.0 mg/L（参考值0～50.0 mg/L），κ/λ比值0.57（参考值1.53～3.29），尿β2微球蛋白0.186 mg/L（参考值0～0.22 mg/L）。骨髓病理示增生较活跃，粒红比例减小，浆细胞比例增高（5％～10％）；骨髓流式细胞术可见少量异常浆细胞，占有核细胞的0.42％。骨髓刚果红染色：可见少量淀粉样物质沉积，刚果红染色弱阳性。最终明确诊断：①获得性凝血因子Ⅹ缺乏症；②获得性凝血因子Ⅴ缺乏症；③pAL；④失血性贫血。评估受累脏器：①心脏：心电图未见肢体导联低电压表现，心脏超声示左心房增大、左心室舒张功能减低。BNP升高，考虑心脏受累。②肝脏：患者有心衰因此无法使用肝总界来判定，碱性磷酸酶158 U/L（未到正常值的3倍），肝功能正常，仅表现为肝大（肋缘下1.5 cm），因此不考虑肝脏受累。③肾脏：有蛋白尿，考虑累及肾脏。综上考虑患者主要受累器官为心脏及肾脏。患者在我院未测血清肌钙蛋白T及NT-proBNP，因此无法确认梅奥2012分期。结合患者肝肾功能及其他预后诊断系统，评估本例患者可能介于1、2期之间。

入院后积极止血治疗，多次输注PCC（每次3000 U）止血治疗后复查腹部CT示血肿逐渐吸收。同时予患者BCD方案（地塞米松20 mg/d，第1、2、4、5、8、9、11、12天；硼替佐米2 mg/d，第1、4、8、11天；环磷酰胺500 mg/d，第1、8、15天）治疗原发病。治疗1个月病情稳定出院，后续治疗于当地医院进行。

## 讨论及文献复习

本病例特点如下：①患者为老年男性；②出血部位为皮肤和腹腔；③查体可见肝肋缘下1.5 cm；④BNP升高，有心衰迹象；⑤骨髓浆细胞比例增高（5～10％）；⑥有蛋白尿且尿免疫固定电泳在γ区可见一条单克隆轻链λ成分；⑦骨髓刚果红染色：可见少量淀粉样物质沉积，呈弱阳性。一般来说有症状的器官或组织活检刚果红染色阳性率>95％，皮下脂肪为75％～80％，骨髓为50％～65％。考虑取材方便，我们在充分预防性止血情况下选择骨髓标本。本例患者未行免疫荧光及质谱法分析，但考虑患者血浆游离轻链水平高，根据临床经验诊断为pAL。

对于凝血功能异常的患者，在排除其他原因导致的凝血功能异常后（如DIC、抗凝药物使用等），应完善APTT混合试验和凝血因子活性测定以确定先天性或获得性凝血因子缺乏。FⅩ缺乏需要考虑以下可能：①先天性凝血因子缺乏症：患者无自幼出血史及相关疾病家族史，无靶关节，各关节功能正常，暂不考虑先天性原因。②针对凝血因子的抗体：患者混合血浆纠正实验阴性，Bethesda法检测FⅩ抑制物定量为0 BU/ml，并且PCC回收试验FⅩ∶C未见提高，因此排除FX抑制物及非中和性抗体存在的可能。③非抗体性凝血因子缺乏症：DIC：患者CDSS评分3分，可排除DIC；肝功能异常：患者肝功能正常，因此暂排除；维生素K缺乏：一般维生素K缺乏会导致维生素K依赖的凝血因子合成障碍（如FⅡ、FⅦ、FⅨ及FX因子缺乏），但患者不存在以上因子活性缺乏，因此暂不考虑该病。PCC回收试验排除了非中和性抗体存在的可能。APTT纠正实验的意义在于鉴别单纯性因子缺乏（先天/合成不良/消耗）及循环抗凝物形成。结合患者凝血功能提示APTT及PT均升高，Bethesda法检测FⅩ抑制物定量为0 BU/ml，且PCC回收率未达到预期，不考虑凝血因子抑制物导致的凝血功能障碍。血免疫固定电泳未见M蛋白，但游离轻链κ和λ增高。尿免疫固定电泳在γ区可见一条单克隆轻链λ成分。骨髓浆细胞比例增高（5％～10％），浆细胞比例<20％，不足以诊断为多发性骨髓瘤。于是对骨髓组织进行了刚果红染色：可见少量淀粉样物质沉积（弱阳性）。患者最终确诊为pAL合并获得性FⅩ、FⅤ缺乏症。

轻链型淀粉样变（AL）导致获得性FⅩ缺乏的病例时有报道，对于此类患者，即使大量输注含FⅩ的新鲜冰冻血浆或PCC也只能极其短暂及有限地提高FⅩ∶C。早在40余年前，Furie等[2]用I^131^标记的FⅩ注入pAL患者体内，发现FⅩ迅速从血液循环中消失，半衰期仅为30 s，24 h内尿液中的排出量也仅为12.6％。全身体表放射性检测发现I^131^遍布全身，肝、脾区存在浓聚。进一步研究发现，从AL淀粉样变患者脾脏分离出淀粉样物质可在体外与FⅩ结合。目前普遍认为，AL淀粉样变患者FⅩ缺乏的发病机制为：凝血因子与淀粉样物质结合，快速从血浆中清除，大量沉积于网状内皮系统（如脾脏），同时淀粉样物质在血管壁的沉积，导致血管壁的脆性增加，是淀粉样变患者出血表现的另一原因。患者临床主要表现为凝血障碍，如自发或咳嗽、呕吐后的眶周瘀斑，颈部及其他易摩擦部位的皮肤瘀斑等。在一项纳入337例AL淀粉样变患者的回顾性研究中，51％的患者存在凝血功能异常，包括TT延长（32％）、PT延长（24％）和APTT延长（14％），其中皮下出血最常见（18％）[3]。导致PT、APTT延长最常见的原因为FⅩ缺乏。AL淀粉样变患者中获得性FⅩ缺乏的发生率为7％～14％。此外，也有获得性FⅤ、Ⅶ、Ⅸ、Ⅻ缺乏的报道。出血的风险及严重程度与FⅩ∶C的相关性暂无定论[4]。

除了pAL，获得性FⅩ缺乏也可继发于慢性淋巴细胞白血病、急性非淋巴细胞白血病、肝硬化、肺部感染、严重烧伤引起的败血症等，有报道称短暂的FX缺乏可能与狼疮抗凝物阳性及抗FⅩ因子抗体形成有关[5]–[11]，但目前具体发病机制仍然不明确。

AL淀粉样变并发获得性FⅩ缺乏症的治疗包括急性出血的替代治疗和原发病的治疗。替代治疗包括输注新鲜冰冻血浆、PCC等，部分文献报道在围术期及急性出血期输注重组人凝血因Ⅶa可取得良好止血效果[12]–[13]。输注血浆及PCC可使患者凝血指标及FⅩ∶C得到一过性改善，但疗效并不明显，对于该病患者，普通替代治疗获益十分有限，应积极治疗原发病。pAL的理想治疗目标是获得器官缓解，但现有的治疗都只是靶向于克隆性浆细胞，降低单克隆免疫球蛋白水平，并最终通过人体自我清除机制获得器官缓解，器官缓解往往发生于血液学缓解3～12个月后[14]–[15]。以往的一项回顾性分析中，7例AL淀粉样变合并FⅩ缺乏的患者接受了以硼替佐米为基础的化疗并进行血液学疗效评估，其中5例获得非常好的部分缓解（VGPR）及以上疗效，4例患者FⅩ∶C提高[16]。推荐治疗方案多来自于多发性骨髓瘤的化疗方案，如BCD（硼替佐米+地塞米松+环磷酰胺）、MP（美法仑+泼尼松）、CTD（环磷酰胺+地塞米松+沙利度胺）、TD（沙利度胺+地塞米松）等。pAL引起的获得性FⅩ缺乏临床少见，因此应加强疾病的认识，避免漏诊。对于存在单克隆免疫球蛋白升高伴出血表现的患者，应考虑pAL的可能。另一方面，无论pAL患者是否存在出血表现，我们都有必要明确其是否存在FⅩ缺乏，以便早期诊断并给予积极有效的治疗，改善预后、延长生存期。
